# The domain organization of the bacterial intermediate filament-like protein crescentin is important for assembly and function

**DOI:** 10.1002/cm.20505

**Published:** 2011-02-28

**Authors:** Matthew T Cabeen, Harald Herrmann, Christine Jacobs-Wagner

**Affiliations:** 1 Department of Molecular, Cellular and Developmental Biology, Yale UniversityNew Haven, Connecticut; 2 Functional Architecture of the Cell Group, German Cancer Research CenterHeidelberg, Germany; 3 Section of Microbial Pathogenesis, Yale School of MedicineNew Haven, Connecticut; 4 Howard Hughes Medical Institute, Yale UniversityNew Haven, Connecticut

**Keywords:** crescentin, coiled-coil, cell curvature, *Caulobacter crescentus*, bacterial cytoskeleton

## Abstract

Crescentin is a bacterial filament-forming protein that exhibits domain organization features found in metazoan intermediate filament (IF) proteins. Structure-function studies of eukaryotic IFs have been hindered by a lack of simple genetic systems and easily quantifiable phenotypes. Here we exploit the characteristic localization of the crescentin structure along the inner curvature of *Caulobacter crescentus* cells and the loss of cell curvature associated with impaired crescentin function to analyze the importance of the domain organization of crescentin. By combining biochemistry and ultrastructural analysis in vitro with cellular localization and functional studies, we show that crescentin requires its distinctive domain organization, and furthermore that different structural elements have distinct structural and functional contributions. The head domain can be functionally subdivided into two subdomains; the first (amino-terminal) is required for function but not assembly, while the second is necessary for structure assembly. The rod domain is similarly required for structure assembly, and the linker L1 appears important to prevent runaway assembly into nonfunctional aggregates. The data also suggest that the stutter and the tail domain have critical functional roles in stabilizing crescentin structures against disassembly by monovalent cations in the cytoplasm. This study suggests that the IF-like behavior of crescentin is a consequence of its domain organization, implying that the IF protein layout is an adaptable cytoskeletal motif, much like the actin and tubulin folds, that is broadly exploited for various functions throughout life from bacteria to humans. © 2011 Wiley-Liss, Inc.

## Introduction

Bacteria have a complex subcellular architecture, a notion that has only recently become apparent thanks to advances in microscopy techniques. We now know that many important cellular functions are performed by counterparts of eukaryotic cytoskeletal proteins [Pogliano,^2008^; Graumann,^2009^; Cabeen and Jacobs-Wagner,^2010^]. For instance, the tubulin homolog FtsZ has an essential role in cell division, whereas actin homologs such as MreB and ParM are important for rod cell shape maintenance and plasmid segregation. The characterization of bacterial tubulin and actin homologs as cytoskeletal proteins has historically followed a common path. Low primary sequence similarity between bacterial and eukaryotic cytoskeletal proteins initially hampered their identification. Instead, the presence of small, structurally significant motifs important for nucleotide binding yielded the first hints that these proteins were cytoskeletal [Bork et al.,^1992^; de Boer et al.,^1992^; RayChaudhuri and Park,^1992^; Mukherjee et al.,^1993^]. Subsequent structural analysis showed that these proteins indeed shared a common fold with eukaryotic tubulin and actin [Löwe and Amos,^1998^; van den Ent et al.,^2001^]. Continued study of bacterial tubulin and actin homologs revealed themes that seem to be hallmarks of bacterial cytoskeletal proteins. In contrast to eukaryotic tubulin and actin, which are extremely conserved even among diverse organisms, bacterial tubulin and actin homologs (e.g., FtsZ or MreB) can vary substantially in sequence across different bacterial species [Erickson,^2007^]. Furthermore, there are several distinct families of tubulin homologs (FtsZ, TubZ, BtubA/B) and a surprisingly large number of actin homolog families in the prokaryotic world. In addition to well-studied actin homologs such as MreB and ParM, recent work uncovered AlfA, with only 17% identity to MreB [Becker et al.,^2006^]. AlfA was then used as a starting point to search for more bacterial actin homologs too divergent in sequence to be identified using MreB as a reference sequence. This approach uncovered as many as 35 additional families of bacterial actin homologs, all with signature actin motifs [Derman et al.,^2009^]. All bacterial tubulin and actin homologs examined so far form filamentous structures in vitro and in vivo [Larsen et al.,^2007^; Derman et al.,^2009^], despite being vastly divergent among themselves and from the canonical eukaryotic sequences. It is clear that bacteria have exploited the flexibility of the core tubulin and actin folds to create proteins with properties tailored to specific functions as diverse as cell shape maintenance, plasmid segregation, and cell division [Cabeen and Jacobs-Wagner,^2010^].

The discovery of bacterial intermediate filament (IF)-like proteins [Ausmees et al.,^2003^; Bagchi et al.,^2008^] further substantiates the notion that core cytoskeletal motifs are adaptable. Eukaryotic IF proteins are typified by their presence in metazoa but not plants, protists or fungi. Two metazoan IF systems can be distinguished: the nuclear lamins, which form a two-dimensional meshwork underlying the inner nuclear membrane, and cytoplasmic IF proteins, which, in humans, represent a diverse group of more than 70 fibrous proteins with various cellular functions. Both lamins and cytoplasmic IF proteins exhibit a core architecture built around a coiled-coil-forming “rod” domain [Herrmann et al.,^2009^]. Most notably, while the N-terminal “head” and C-terminal “tail” domains can differ substantially in length, sequence, and structure, the rod domain always displays an arrangement of distinct coiled-coil motifs of largely conserved length separated by short linkers [Herrmann et al.,^2009^]. The fibrous nature of IF proteins makes full-length proteins refractory to crystallization, but their shared coiled-coil domain architecture, and crystallography of protein fragments [Strelkov et al.,^2004^], indicates that they all fold into a similar three-dimensional coiled-coil structure [Herrmann et al.,^2009^].

The founding member of the bacterial IF-like family, crescentin, forms a single filamentous structure along the inner curvature of the crescent-shaped bacterium *Caulobacter crescentus* [Ausmees et al.,^2003^; Cabeen et al.,^2009^]. Without crescentin, *C. crescentus* cells lose their curvature and become straight rods [Ausmees et al.,^2003^]. Similar to IFs [Yoon et al.,^2001^], the crescentin structure is very stable in vivo, displaying little to no subunit turnover along its length [Charbon et al.,^2009^; Esue et al.,^2010^]. New crescentin molecules are incorporated all along the length of the existing structure [Charbon et al.,^2009^]. In vitro, His-tagged crescentin forms IF-like filaments [Ausmees et al.,^2003^], and the rheological properties of purified crescentin solutions resemble those of filament networks composed of the eukaryotic IF vimentin [Esue et al.,^2010^]. The current model for how the crescentin structure works in vivo proposes that the crescentin structure resists strain (a property characteristic of IF) produced by cell elongation and thereby mechanically constrains cell wall synthesis to impart cell curvature [Cabeen et al.,^2009^; Jiang and Sun,^2010^].

**Fig 1:**
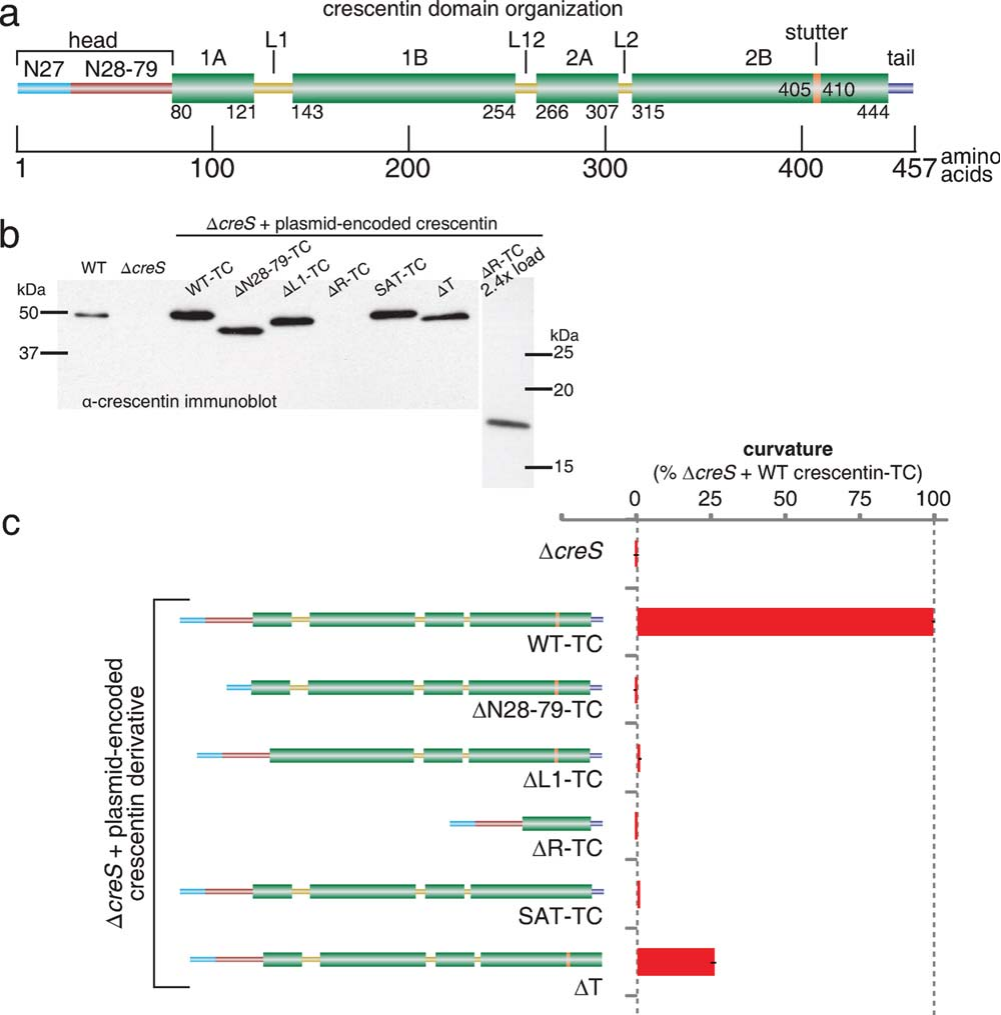
Functional assessment of crescentin mutants in cell curvature. (a) Schematic of crescentin domain organization. Coiled coil-forming regions are indicated by thick green bars and labeled by amino acid positions at junctions with other features; linkers L1, L12, and L2; N27 and N28-79 subdomains of the head, tail, and stutter are labeled. (b) Western blot of crescentin mutants using α-crescentin. The relatively weak signal of crescentin_ΔR_ is likely due to epitope loss. Loading was normalized by OD_660_. (c) The ability of the deletion mutants to curve *C. crescentus* Δ*creS* cells is shown. Curvature values relative to Δ*creS* cells producing wild-type crescentin-TC from the same low-copy plasmid pMR20 (100%). Error bars represent the standard error of the mean (SEM) of three experiments; each experiment examined, on average, over 1200 cells.

At the sequence level, crescentin is more similar to eukaryotic IF proteins (such as cytokeratin 19, with ∼ 25% identity and 40% similarity; [Ausmees et al.,^2003^]) than MreB or FtsZ is to actin or tubulin [Erickson,^2007^], although this is not entirely surprising given the repeated nature and the occurrence of the coiled-coil motif in both crescentin and IF proteins. Crescentin also shares the typical tripartite IF-like domain architecture, with a coiled-coil rod domain interrupted by short linker sequences and flanked by short head and tail domains [Ausmees et al.,^2003^] (Fig. 1a). Even a stutter, a small interruption in the characteristic coiled-coil heptad repeat pattern in coil 2 of eukaryotic IF proteins [Herrmann et al.,^2009^], is present in crescentin [Ausmees et al.,^2003^] (Fig. 1a). However, crescentin lacks the conserved “IF-consensus motifs” at either end of the rod domain, although a few eukaryotic IF proteins, such as phakinin and filensin, diverge substantially from the consensus [Herrmann et al.,^2000^,^2009^]. Importantly, it is unclear whether the IF-like protein organization of crescentin is important for crescentin function.

Several mutagenesis studies on eukaryotic IF proteins have examined the roles of specific regions (e.g., head, linker L1, stutter, and tail) in IF assembly in vitro using purified proteins. In some cases, in vivo assembly was examined by looking at the ability of mutated proteins to incorporate into (or disrupt) the pre-existing IF network in cell lines, or to form filaments in IF-free cell lines. However, structure-function relationship studies have generally been limited to known disease-related point mutations [Bär et al.,^2005^,^2006^,^2010^], most likely because of the lack of good functional assays in cultured cells. Cells lacking cytoplasmic IF proteins do not exhibit any discernable defects in growth, colony-formation, or morphology [Venetianer et al.,^1983^; Hedberg and Chen,^1986^]. As for nuclear lamins, only dominant effects of mutant proteins on the endogenous lamins have typically been examined [Spann et al.,^1997^; Schirmer et al.,^2001^]. Functional studies are complicated by some IF proteins being essential for viability (e.g., lamins), the existence of redundancies among IF proteins (e.g., keratins) or the complexity of mutant phenotypes at the tissue or organismal level [Harborth et al.,^2001^; Zhou et al.,^2003^; Herrmann et al.,^2007^]. In contrast, the characteristic localization of crescentin along the inner curvature of *C. crescentus* cells and the loss of cell curvature associated with a loss of crescentin function provide quantifiable assays for in vivo functional studies [Cabeen et al.,^2009^]. We use this bacterial model system to investigate two questions. Are the IF-like organizational features of crescentin functionally important, as opposed to merely incidental? Do different features have distinct roles in creating a functional crescentin structure?

## Materials and Methods

### Bacterial Culture, Strains, Plasmids, and Mutagenesis

*C. crescentus* was grown in peptone-yeast extract (PYE) or M2G supplemented with 1% PYE (M2G^+^) at 30°C [Ely,^1991^]. Plasmids were transformed into *C. crescentus* by electroporation or conjugation with *Escherichia coli* strain S17-1 [Ely,^1991^]. Strains and plasmids are listed in Table I, and their modes of construction and primer sequences are given in the Supporting Information. Exponentially growing cultures were used for all experiments.

**Table I:** Strains and Plasmids Used in This Work

Strain	Relevant genotype or description	Reference or source
*C. crescentus*		
CB15N	Synchronizable variant strain of CB15; also known as NA1000	Evinger and Agabian,^1977^
CJW932	CB15N *creS*::pJM21creS-flag	Ausmees et al.,^2003^
CJW1231	CB15N/pMR20creS-tc	This study
CJW1233	CB15N Δ*creS*/pMR20creS-tc	Cabeen et al.,^2009^
CJW1474	CB15N/pMR20creSΔT	This study
CJW1475	CB15N Δ*creS*/pMR20creSΔT	This study
CJW1478	CB15N/pMR20creSΔR-tc	This study
CJW1518	CB15N Δ*creS*/pMR20creSSAT-tc	This study
CJW1520	CB15N/pMR20creSΔL1-tc	This study
CJW1521	CB15N Δ*creS*/pMR20creSΔL1-tc	Cabeen et al.,^2009^
CJW1522	CB15N/pMR20creSSAT-tc	This study
CJW1535	CB15N/pMR20creSΔN28-79-tc	This study
CJW1536	CB15N Δ*creS*/pMR20creSΔN28-79-tc	This study
CJW2862	CB15N Δ*creS*/pMR20creSΔR-tc	This study
CJW2927	CB15N Δ*creS*/pMR20creSΔN27-tc	Cabeen et al.,^2009^
LS3812	CB15N Δ*creS*	Gitai et al.,^2004^
*E. coli*		
DH5α	Cloning strain	Invitrogen
S17-1	RP4-2 *Tc*::*MuKM*-Tn*7*, for plasmid mobilization	Simon et al.,^1983^
BL21 (DE3)	F^-^*ompT hsdS*_B_ (rB^-^ mB^-^) *dcm gal* (DE3)	Novagen
CJW1659	BL21 (DE3)/pET29acreS	This study
CJW1353	BL21 (DE3)/pET29acreSΔN27	This study
CJW2054	BL21 (DE3)/pET29acreSΔL1	This study
CJW2045	BL21 (DE3)/pET29acreSSAT	This study
CJW3663	BL21 (DE3)/pET29acreSΔT	This study
Plasmid	Description	Reference or Source
pBluescriptKS+	AmpR cloning vector	Stratagene
pET29a	Protein expression plasmid	Novagen
pET29acreS	pET29a carrying *creS* coding sequence	This study
pET29acreSΔN27	pET29a carrying *creS*_Δ*N27*_ coding sequence	This study
pET29acreSΔL1	pET29a carrying *creS*_*ΔL1*_ coding sequence	This study
pET29acreSSAT	pET29a carrying *creS*_*SAT*_ coding sequence	This study
pET29acreSΔT	pET29a carrying *creS*_Δ*T*_ coding sequence	This study
pHL23	KanR cloning vector	Lam et al.,^2006^
pMR20	TetR low-copy-number broad host range vector	Roberts et al.,^1996^
pMR20creS-tc	pMR20 carrying *creS-tc* gene, coding for crescentin with a C-terminal tetracysteine tag	Cabeen et al.,^2009^
pMR20creSΔN28-79-tc	pMR20 carrying *creS-tc* gene deleted for N28-79 coding sequence	This study
pMR20creSΔL1-tc	pMR20 carrying *creS-tc* ORF deleted for linker 1 coding sequence	Cabeen et al.,^2009^
pMR20creSΔR-tc	pMR20 carrying *creS-tc* gene deleted for rod coding sequence	This study
pMR20creSΔT	pMR20 carrying *creS* gene deleted for tail coding sequence	This study
pMR20creSSAT-tc	pMR20 carrying *creS-tc* gene with coding sequence for Ser-Ala-Thr inserted at stutter site	This study

### Light Microscopy and Cell Curvature Analysis

Cells were imaged at room temperature (∼ 22°C) on a Nikon E1000 microscope fitted with 100× differential interference contrast or phase-contrast objectives and a Hamamatsu Orca-ER LCD camera, or a Nikon E80i microscope fitted with similar objectives and a Hamamatsu Orca-IIER LCD camera. Cells were immobilized on 1% agarose-PYE or M2G^+^ pads. FlAsH staining of tetracysteine (TC)-tagged crescentin was performed as previously described [Cabeen et al.,^2009^]. Immunofluorescence microscopy was performed as described [Ausmees et al.,^2003^] using anti-crescentin antibodies at 1:200. Image capture and analysis were performed with MetaMorph software (Molecular Devices). Cell curvature analysis was performed on phase-contrast images as described [Cabeen et al.,^2009^,^2010^]. Three separate experiments were performed for each strain, and the number of cells analyzed for each experiment ranged from 633 to 2150. Statistical calculations were performed with MATLAB and Microsoft Excel.

### Immunoblotting

Cell extracts (load volumes normalized by culture optical density at 660 nm; OD_660_) were separated on sodium dodecyl sulfate (SDS)-10% polyacrylamide gels, transferred to Immobilon P membranes (Millipore) using a Bio-Rad Trans-Blot apparatus, and blotted with affinity-purified anti-crescentin antibodies at 1:10,000. Quantitative immunoblotting was performed by comparing a standard dilution series of purified crescentin as standards to wild-type (CB15N) cell extracts from known quantities of cells. Chemiluminescently stained blots were analyzed on a Typhoon scanner (GE) and quantified using ImageQuant.

### Protein Purification

Untagged wild-type and mutant versions of crescentin were purified under identical conditions from *E. coli* BL21 (DE3) strains carrying the *creS* (or mutant) coding sequence in pET29a. Cells (0.5–1 L) were grown at 37°C in LB medium to OD_600_ ∼ 0.6, then induced with 1 mM isopropyl β-D-1-thiogalactopyranoside for 3 h. Cells were harvested by centrifugation at 3000 × *g* for 15 min at 4°C and frozen at −80°C overnight. Thawed cells were resuspended and lysed in Buffer A (25 mM sodium acetate pH 4.5, 8 M urea) and the lysate was cleared at 12,000 × *g* for 30 min. Urea was always freshly prepared in the presence of a mixed-bed ion-exchange resin. Wild-type and mutant versions of crescentin were purified from the lysate on CM-sepharose (GE) equilibrated with Buffer A, washed with Buffer A, and eluted with a salt gradient (0–300 mM or 0–150 mM NaCl in Buffer A). Crescentin-containing fractions [determined by SDS-polyacrylamide gel electrophoresis (PAGE)] were pooled and the urea was removed by 15 min-stepwise dialysis (25 kDa MWCO), halving the urea concentration each time, then overnight dialysis against 2 L pure 5 mM HEPES pH 7.5 or Tris-HCl pH 8.5 (dialysis at pH 6.5 caused precipitation). Crescentin and crescentin mutant concentration was determined using a Bradford assay (Bio-Rad). Protein preparations were stored in buffer at 4°C and checked for protein degradation by PAGE before use.

### Pelleting Assays

Crescentin samples (20 μL) at ∼ 8 μM were mixed 1:1 with a 2× “assembly buffer” composed of buffer (PIPES pH 6.5, HEPES pH 7.5, or Tris-HCl pH 8.5) and salt (KCl or MgCl_2_) as noted; samples at pH 6.5 were kept at 5.4 μM to preserve solubility and mixed 3:1 with a 4× assembly buffer. The 40 μL final samples at 4 μM were then allowed to polymerize for 10–15 min at room temperature before pelleting at 135,500 × *g* for 15 min at 25°C (Beckman Optima Max-XP, TLA-100 rotor). Supernatant samples (16 μL) were taken from the top of the tube, remaining supernatant was removed, and pelleted material was solubilized in 40 μL 5 mM Tris-HCl pH 8.5, 8 M urea for ∼ 10 min. A 16 μL sample of the solubilized pellet was then taken. The supernatant and pellet fractions were mixed 4:1 with 5× SDS-PAGE loading buffer, run on 10% polyacrylamide gels, stained with Coomassie Brilliant Blue, and digitally scanned using a flatbed scanner. Relative protein amounts in the supernatant and pellet fractions were determined by densitometry of inverted images using ImageJ. All experiments were performed in triplicate. Loading known amounts of crescentin confirmed the accuracy of the densitometry method.

### Electron Microscopy

Samples of crescentin preparations were diluted to 4 μM with 2× or 4× assembly buffer as described above, allowed to polymerize for 10–15 min at room temperature, and applied to freshly glow-discharged 400 mesh carbon-coated copper grids (Electron Microscopy Sciences). After allowing proteins to settle for 2–3 min, grids were washed with water three times (water was removed after the last wash by touching the grid to filter paper) and stained for about 1 min with 1% uranyl acetate. The uranyl acetate was removed using filter paper and the grids were dried for several hours. The samples were viewed with a JEOL JEM-1230 transmission electron microscope at 80 kV and images were acquired with a Hamamatsu ORCA-HR digital camera.

## Results

### Domain Mutants are Impaired in Cell Curvature Function

Previous mutagenesis studies on eukaryotic IF proteins have shed light on the role of some elements of the conserved IF domain organization in filament assembly (as described below). We combined this knowledge to examine in a single study whether several analogous mutations in crescentin affect its function in vivo. Each mutant, with the exception of the tail mutant (crescentin_ΔT_; see below), was tagged with a TC tag for visualization inside cells using the fluorescent dye FlAsH [Griffin et al.,^1998^] since TC tagging, unlike GFP tagging, does not disrupt crescentin function [Ausmees et al.,^2003^; Cabeen et al.,^2009^]. All mutants were produced from a low-copy plasmid under the native promoter in a crescentin-null background as the only source of crescentin in the cell. Production of each mutant was confirmed by anti-crescentin Western blotting, with the less intense staining of the rod mutant (crescentin_ΔR_) likely due to epitope loss from the large deletion (Fig. 1b). We used automated cell curvature analysis to assess the function of each mutant (Fig. 1c).

The head domain of eukaryotic IF proteins, despite high variability in length and amino acid sequence, is generally regarded as essential for IF function. The head is required for polymerization of both nuclear [Heitlinger et al.,^1992^] and cytoplasmic IF beyond oligomeric subunits in vitro [Hatzfeld and Burba,^1994^; Herrmann et al.,^1996^] and for filament formation in vivo [Raats et al.,^1990^; Beuttenmüller et al.,^1994^; Spann et al.,^1997^]. Furthermore, phosphorylation or point mutations in the head adversely affect self-assembly [Geisler and Weber,^1988^; Sharma et al.,^2009^]. We therefore analyzed the function of the head domain of crescentin in *C. crescentus* curvature. Importantly, we distinguished between two subdomains in the crescentin head. We had previously shown that the amino-terminal N27 subdomain (Fig. 1a) is not necessary for crescentin structure formation in vivo but rather is required for the association of the crescentin structure with the cell wall and hence cell curvature [Cabeen et al.,^2009^]. Here, we generated an in-frame deletion that maintains the N27 region but removes the remainder of the head (Fig. 1a), and analyzed the ability of the resulting mutant protein (termed crescentin_ΔN28-79_) to mediate cell curvature. Cells producing crescentin_ΔN28-79_-TC displayed no detectable curvature (Fig. 1c), indicating that this deletion completely abrogated crescentin function.

In vimentin, a functional deletion of linker L1 yields a protein able to assemble beyond small oligomers into amorphous aggregates but unable to elongate into filaments in vitro [Herrmann et al.,^1999^]. The short, 8-residue length of linker L1 in vimentin was effectively transformed into part of the rod by the addition of three residues that restore a coiled-coil sequence [Herrmann et al.,^1999^]. Given the longer length of crescentin L1 (21 residues), we utilized a deletion mutant (Δ122-142) lacking L1 and fusing coils 1A and 1B to each other. We had previously reported that this crescentin_ΔL1_ mutant was nonfunctional and in fact had substantial dominant-negative activity [Cabeen et al.,^2009^]; however, we had not quantitatively analyzed the curvature of a strain producing crescentin_ΔL1_-TC. This analysis confirmed that crescentin_ΔL1_-TC bears no measurable cell curvature function (Fig. 1c).

The rod domain, with its coiled-coil-forming regions, would be predicted to have a key role in assembly. Few studies have analyzed the role of the rod domain, but nuclear lamin B1 deleted for all but the first and last five coiled-coil heptad repeats in the rod domain resulted in a dominant-negative effect, causing a nuclear morphology defect in cultured cells [Schirmer et al.,^2001^]. As lamin B1 is essential for viability [Harborth et al.,^2001^], the functional properties of rod-deleted lamin B1 alone have not been examined. We generated an analogous deletion of all but the first and last 5 coiled-coil heptads in the rod domain of crescentin. Consistent with an important functional role, crescentin_ΔR_-TC induced no detectable cell curvature (Fig. 1c).

Removal of the coil 2B stutter in vimentin produced a protein capable of the initial stages of assembly but defective in filament elongation in vitro, suggesting that the stutter plays an important role in filament assembly [Herrmann et al.,^1999^]. In vimentin, stutter removal was accomplished by the insertion of three amino acids (serine, alanine, and threonine; S-A-T) that restored the coiled-coil heptad repeat pattern [Herrmann et al.,^1999^]. We employed the same strategy, as a S-A-T insertion in crescentin restores the coiled-coil heptad repeat pattern and eliminates the stutter, according to the MARCOIL prediction algorithm [Delorenzi and Speed,^2002^]. Cells producing the resulting crescentin_SAT_-TC showed no measurable curvature (Fig. 1c), suggesting that the stutter in crescentin is functionally important as well.

Finally, we created a C-terminal truncation of crescentin that lacked the tail region. The predicted tail region of crescentin is very short and α-helical. In vimentin, deletion of the tail resulted in greater variation in filament width in vitro, and thicker filaments in cells, compared to the wild-type [Herrmann et al.,^1996^]. However, tail-deleted vimentin could still form filament networks in transfected cells [Rogers et al.,^1995^], consistent with a minor role for the tail in assembly. Crescentin_ΔT_ lacked 13 C-terminal amino acids, and we chose not to TC-tag crescentin_ΔT_ in order to avoid generating an artificial C-terminal tail. Crescentin_ΔT_, unlike the other crescentin mutants, was able to support some curvature. However, cells producing crescentin_ΔT_ displayed on average only a fraction of the curvature (26%) of an isogenic strain producing wild-type crescentin-TC (Fig. 1c), suggesting a substantial defect in cell curvature function. The fact that crescentin_ΔT_ was the only mutant to retain some function is consistent with the tail playing a less crucial role, as observed with eukaryotic IF proteins. On the other hand, the head, linker L1, rod, and stutter are essential for crescentin function in vivo, consistent with eukaryotic IF studies.

### Domain Mutants Interfere with the Function of Wild-Type Crescentin

**Fig 2:**
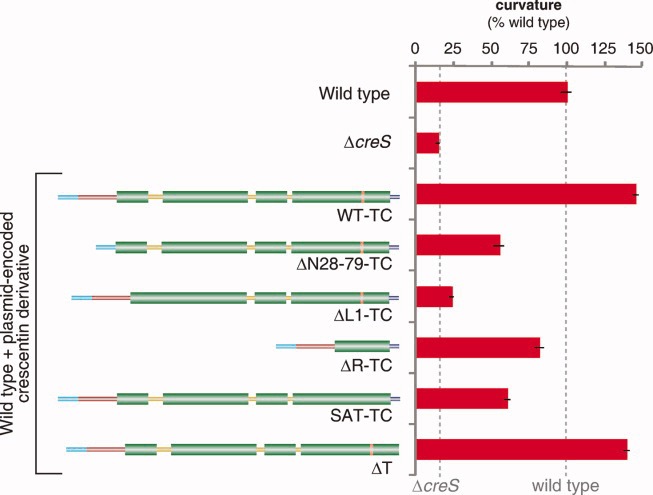
Cell curvature effect of crescentin mutants in the presence of wild-type crescentin. Plasmid-encoded crescentin or crescentin mutants were produced in a background (CB15N) producing wild-type crescentin from the chromosome and were assessed for their effect on cell curvature. The curvature values are relative to wild-type CB15N cells (lacking plasmid). Error bars represent SEM of three experiments; each experiment examined, on average, over 1200 cells.

Production of rod-deleted lamin B1 in cultured cells, or microinjection of head-deleted lamin A into *Xenopus* oocytes, negatively affects the localization and function of wild-type lamins [Spann et al.,^1997^; Schirmer et al.,^2001^]. Such dominant-negative activity is likely driven by formation of disruptive heterocomplexes between the mutant and wild-type proteins and thus informs about the interaction strength between wild-type and mutant proteins. Do crescentin mutants have a similar negative effect on wild-type crescentin? To address this question, we introduced plasmid-encoded crescentin-TC mutants into the wild-type CB15N background producing wild-type crescentin from the chromosome. For comparison, production of wild-type crescentin-TC from the same low-copy-number plasmid resulted in a small but quantifiable hypercurvature relative to the wild-type CB15N lacking plasmid (Fig. 2), consistent with overproduction leading to cell hypercurvature [Cabeen et al.,^2009^]. In contrast, production of four of the five crescentin mutants resulted in decreased curvature values (Fig. 2), showing that they possess dominant-negative activity. Crescentin_ΔL1_-TC, which has previously been reported as a potent dominant negative [Cabeen et al.,^2009^], had the strongest effect with a near-complete abrogation of curvature. Crescentin_ΔN28-79_-TC and crescentin_SAT_-TC both had an intermediate effect, reducing curvature values to roughly half of wild-type, while crescentin_ΔR_-TC had the mildest negative effect, slightly reducing curvature from the wild-type value. These results show that despite their lack of function when expressed on their own, these crescentin mutants are not inert. Rather, they appear to retain the ability to interact with wild-type crescentin to affect its function. The milder effect of crescentin_ΔR_-TC is consistent with the idea that disruption occurs through heterocomplex formation, as interactions between wild-type crescentin and crescentin_ΔR_-TC are expected to be weakened by the much shorter coiled-coil region of the rod-deleted mutant. Conversely, the longer uninterrupted coiled-coil of crescentin_ΔL1_-TC might strengthen heterocomplexes, leading to its strong disruptive effect.

Interestingly, crescentin_ΔT_, which on its own could support only a small fraction of wild-type curvature function (Fig. 1c), was able to induce cell hypercurvature at nearly the level of wild-type crescentin when produced in combination with endogenous crescentin (Fig. 2). This result suggests that crescentin_ΔT_, while deficient in establishing a functional structure on its own, can “strengthen” a wild-type crescentin structure and enhance cell curvature. In this case, heterocomplexes between crescentin_ΔT_ and wild-type crescentin may be functional rather than disruptive.

### Localization of Crescentin Mutants

**Fig 3:**
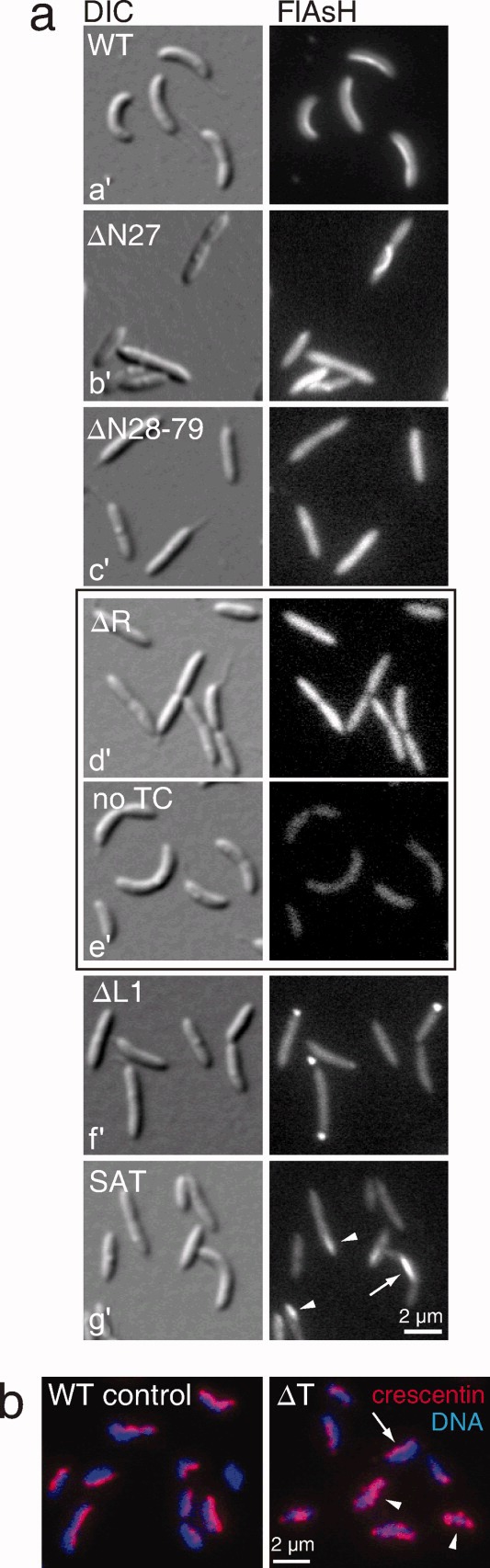
Localization of crescentin domain mutants. (a) Differential interference contrast (DIC) and fluorescence micrographs of FlAsH-stained cells producing TC-tagged wild-type or mutant crescentin proteins from a low-copy plasmid in a crescentin-null Δ*creS* background. For crescentin_ΔR_(d′), which displayed the weakest staining, a CB15N control (i.e., no TC tag), stained, imaged, and scaled identically to the ΔR mutant, is shown (e′). The arrow in (g′) shows a curved cell with a bright crescentin_SAT_-TC structure, and arrowheads indicate cells with short, dimly stained structures. (b) Anti-crescentin immunofluorescence of crescentin_ΔT_, which is untagged in order to prevent the generation of an artificial tail. The arrow indicates a cell with a wild-type staining pattern and arrowheads indicate cells with aberrant localization. A wild-type control, stained in parallel, is given at left for comparison.

The crescentin mutants all displayed defects in cell curvature (Fig. 1c). Could localization defects be responsible for these impairments? Wild-type crescentin-TC stained with FlAsH displayed the expected filament-like localization along the inner curvature of *C. crescentus* cells (Figs. 3a-a′). Each of the crescentin mutants, meanwhile, had localization defects ranging from mild to severe. Crescentin_ΔN27_-TC formed curved, detached filamentous structures in some cells [Cabeen et al.,^2009^], while many cells displayed a diffuse localization (Fig. 3a-b′). Both crescentin_ΔN28-79_-TC and crescentin_ΔR_-TC gave diffuse cytoplasmic signals (Fig. 3a-c′ and 3a-d′, respectively), suggesting a defect in filamentous structure assembly. The diffuse fluorescence of crescentin_ΔR_-TC, the weakest among the mutants, was more intense than background fluorescence of FlAsH-stained wild-type CB15N cells producing no TC-tagged protein (Fig. 3a-e′), arguing that it represents a true localization pattern for crescentin_ΔN28-79_-TC and crescentin_ΔR_-TC. Crescentin_ΔL1_-TC frequently displayed fluorescent foci, as previously reported [Cabeen et al.,^2009^]; shown in Fig. 3a-f′ for comparison, in agreement with an aggregative phenotype wherein crescentin_ΔL1_-TC molecules form clusters.

Crescentin_SAT_-TC was characterized by three distinct localization patterns. About half the cells (49.5%, *n* = 384) showed only diffuse fluorescence, while most other cells (47.9%) contained short, dimly staining crescentin structures that had no evident ability to induce cell curvature (Fig. 3a-g′, arrowheads). The remaining small fraction of cells (2.6%) displayed curvature that was associated with short, brightly staining crescentin_SAT_-TC structures at the site of curvature (Fig. 3-g′, arrow). The curvature of these cells often appeared highly localized, with a sharp curve at the location of the crescentin_SAT_-TC structure. The low frequency of curved cells and the short appearance of the crescentin_SAT_-TC structures suggest that crescentin_SAT_-TC is partially defective both in forming full-length filamentous structures and in mediating cell curvature.

Since crescentin_ΔT_ was not TC-tagged, we used anti-crescentin immunofluorescence microscopy to visualize this mutant. Unlike wild-type crescentin (Fig. 3b), crescentin_ΔT_ failed to produce a consistent filament-like localization. Instead, it displayed multiple patterns ranging from wild-type localization (Fig. 3b, arrow) to spotty, punctate localization patterns (Fig. 3b, arrowheads). This variable localization is consistent with the low median curvature of this strain population, as cells with a more severe protein localization defect would display straighter morphology and cells with a more wild-type localization would display greater curvature.

Our results show that different crescentin mutants display localization defects that differ in severity. The diffuse localization patterns of crescentin_ΔN28-79_ and crescentin_ΔR_ are consistent with no visible assembly whatsoever within cells. The rest of the mutants, however, show some form of crescentin structure (short filaments, detached filaments, foci or puncta) in at least some cells. We analyzed these structure-forming crescentin mutants in vitro to determine whether their aberrant localization patterns in cells might be related to structural or assembly defects inherent in the mutant crescentin proteins themselves.

### Solubility and Ultrastructural Analysis of Wild-Type Crescentin

In order to analyze the in vitro properties of structure-forming crescentin mutants, we first needed to characterize wild-type crescentin as a comparison. We overproduced and purified recombinant crescentin from *E. coli* cells (see Materials and Methods); no protein tag was used for purification so not to affect the sequence and assembly properties of crescentin. We then first determined the assembly properties of wild-type, untagged crescentin using a solubility assay. For this assay as well as for the electron microscopy analysis described below, we used a protein concentration of 4 μM. Quantitative immunoblotting yielded estimates of crescentin abundance at 500–1000 molecules per cell, which corresponds to 2.1–4.2 μM (assuming cylindrical cells of 2 μm length and 0.5 μm diameter).

Cytoplasmic IF proteins such as vimentin and keratins are characterized by solubility in buffers of high pH and low ionic strength, while lower pH and higher ionic strength strongly favor filament assembly and hence insolubility [Herrmann and Aebi,^2004^]. We therefore varied the buffer strength at pH values of 6.5, 7.5, and 8.5 and probed the effects of monovalent K^+^ and divalent Mg^2+^ cations on crescentin assembly. To test the solubility of crescentin under various conditions, we employed an ultracentrifugation-based pelleting assay. The portion of the protein sample found in the pellet was considered as the insoluble fraction.

**Fig 4:**
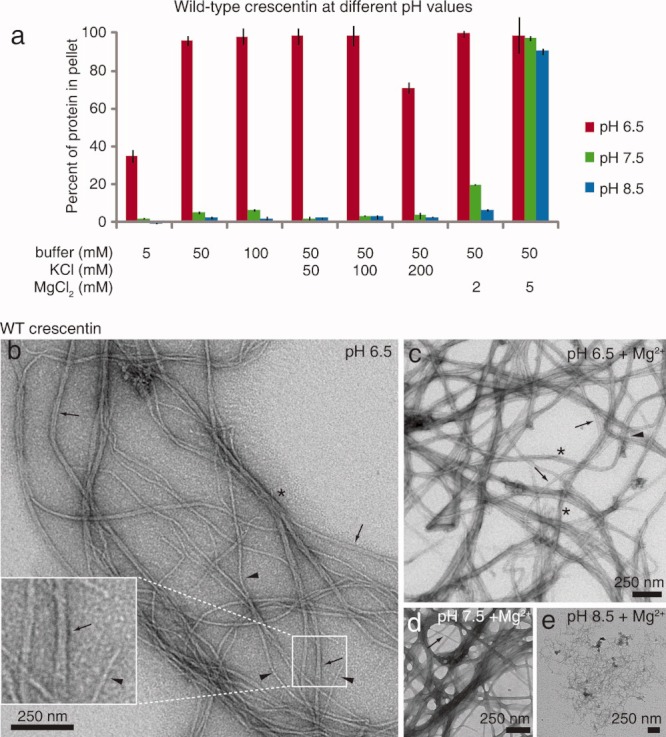
Wild-type crescentin solubility and ultrastructure. (a) Solubility of purified wild-type crescentin over a range of pH values, buffer concentrations, and KCl and MgCl_2_ concentrations. Bars show the proportion of crescentin pelleted after centrifugation for 15 min at 135,000 × *g*. The average of three experiments is shown, and error bars represent SEM. (b–e) Transmission electron micrographs of negatively stained wild-type crescentin. Samples (0.2 mg/mL, ∼ 4 μM) were in 50 mM PIPES (pH 6.5), HEPES (pH 7.5), or Tris-HCl (pH 8.5). Mg^2+^ denotes 5 mM MgCl_2_. (b) Single filaments of 8–10 nm width (arrowheads), presumed pairs with widths of 17–20 nm (arrows), and a 50 nm-wide bundle (asterisk) are denoted. (c) Crescentin sample in the presence of 5 mM Mg^2+^. A single 10 nm-wide filament (arrowhead) and 20–50 nm (arrows and asterisks, respectively) bundles are denoted. (d) Crescentin sample in the presence of 5 mM Mg^2+^ at pH 7.5. A 20 nm-wide structure is denoted with an arrow. (e) Wild-type crescentin at pH 8.5 in the presence of 5mM Mg^2+^. Ten nanometer and wider filaments compose the finely reticulated network.

Similar to eukaryotic IF proteins, wild-type crescentin assembly showed a dependence on pH and ionic strength, with lower pH and higher ionic strength strongly favoring assembly (Fig. 4a). Wild-type crescentin was nearly completely soluble at pH 7.5 and 8.5, and assembly at these pH values could only be induced by the presence of 5 mM Mg^2+^ ions. At pH 6.5, meanwhile, crescentin was highly insoluble except in buffer of very weak ionic strength (5 mM). Monovalent K^+^ cations had little effect on solubility except at the highest concentration tested (200 mM), where they increased solubility slightly (Fig. 4a).

Our pelleting assays measured insolubility rather than giving data about the size or shape of the insoluble crescentin material. To examine the structures formed, we used transmission electron microscopy (TEM) of negatively stained crescentin samples. Crescentin formed IF-like filaments of 8–10 nm width at pH 6.5 (Fig. 4b, arrowheads) as well as 17–20 nm-wide filament pairs (Fig. 4b, arrows) or larger bundles (∼ 50 nm, Fig. 4b, asterisk), indicating a propensity for lateral association. Filaments were also readily visible in five-fold diluted crescentin samples (0.8 μM; data not shown). Addition of 5 mM MgCl_2_ at pH 6.5 increased the appearance of 20 nm-wide pairs (Fig. 4c, arrows) and larger bundles (Fig. 4c, asterisks), consistent with the divalent Mg^2+^ ions having a bundling effect. Wider filamentous structures in the presence of Mg^2+^ were also predominant at pH 7.5 (Fig. 4d), while at pH 8.5, finely reticulated filament networks were most commonly observed (Fig. 4e). Crescentin filaments were also bent along their length, thereby resembling the characteristic flexibility of eukaryotic IF proteins [Herrmann et al.,^2007^].

### Solubility and Ultrastructural Analysis of Crescentin Mutants

We next purified each (tag-less) crescentin mutant that formed some sort of structures visible by fluorescence microscopy in vivo (Fig. 3) and analyzed their solubility under a subset of the conditions used for wild-type crescentin. We also included crescentin_ΔN27_ in this study, which forms an intracellular filamentous structure [Cabeen et al.,^2009^,^2010^] but has not yet been studied in vitro. We varied pH over the same range of buffer concentrations and tested the effects of K^+^ and Mg^2+^ ions at their highest concentrations (200 mM and 5 mM, respectively), where they exerted the clearest effect on wild-type crescentin solubility.

**Fig 5:**
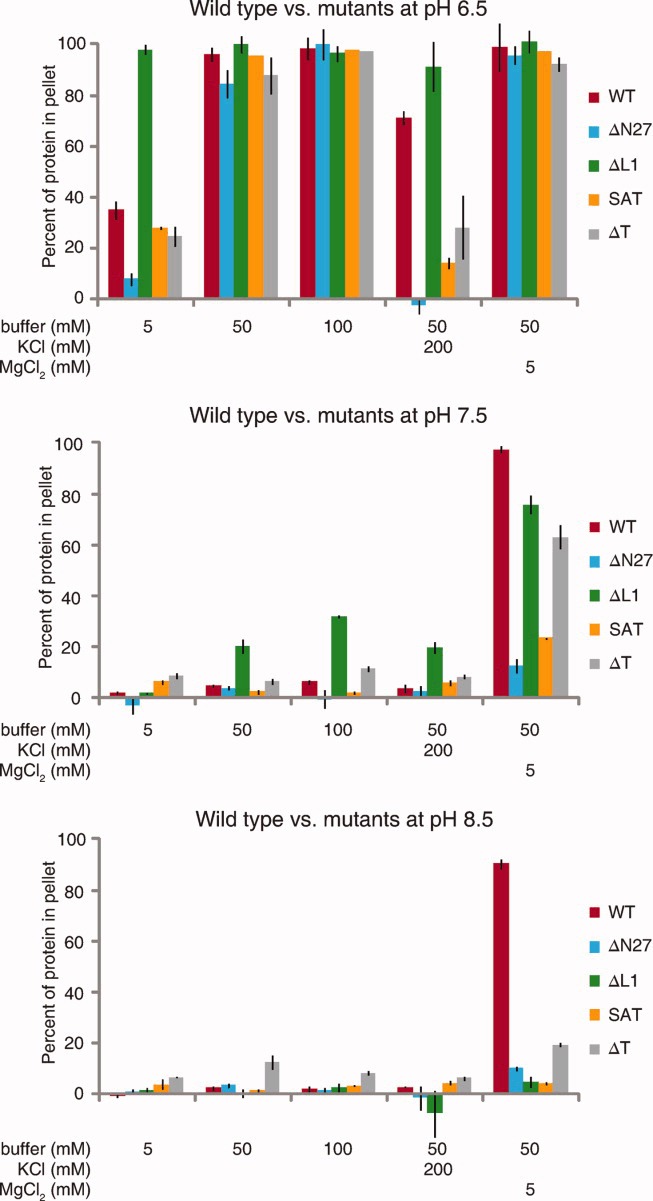
Solubility of crescentin mutants. Comparison of wild-type and mutant solubility at pH 6.5, 7.5, and 8.5 under the noted conditions. Bars show the proportion of crescentin pelleted after centrifugation for 15 min at 135,000 × *g*. The average of three experiments is shown, and error bars represent SEM.

As with wild-type crescentin, lower pH strongly favored pelleting of all the crescentin mutants, with significant assembly at pH 7.5 and 8.5 occurring only in the presence of 5 mM Mg^2+^ ions (Fig. 5). Strikingly, crescentin_ΔL1_ was more insoluble than wild-type crescentin or any of the other mutants in many conditions. It was nearly completely insoluble across all conditions at pH 6.5, even in weak buffer (5 mM) in the absence of monovalent or divalent cations (Fig. 5). Crescentin_ΔL1_ also showed slightly enhanced insolubility at pH 7.5 relative to wild-type and the other mutants. The high insolubility of crescentin_ΔL1_ implies strong assembly or aggregation that is consistent with the compact, focus-like localization (Fig. 3a-f′) and severe dominant-negative effect of crescentin_ΔL1_ in cells (Fig. 2).

**Fig 6:**
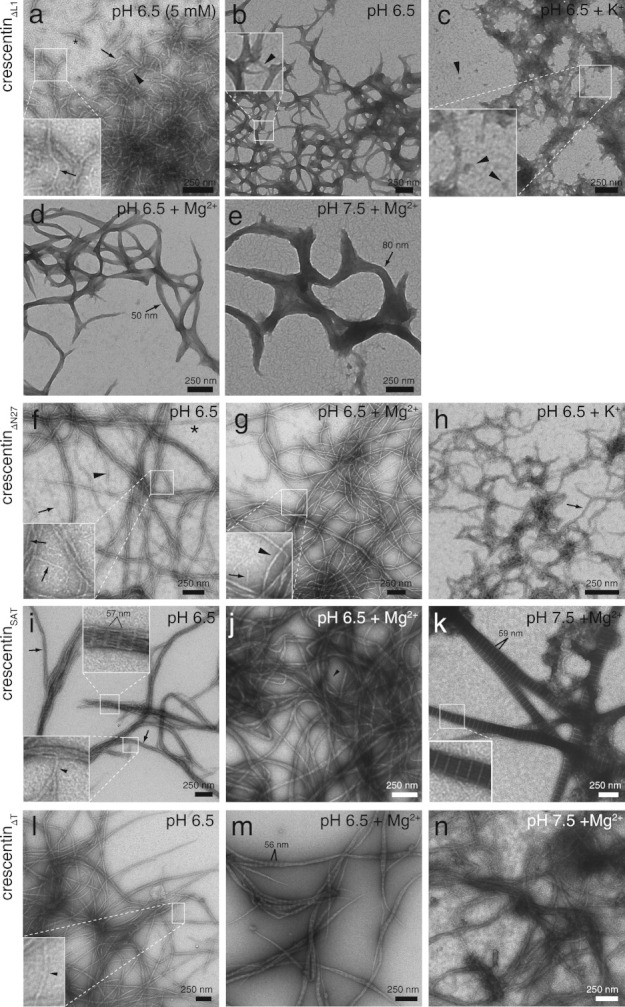
Ultrastructure of negatively stained crescentin mutants. Samples of purified crescentin mutants (0.2 mg/mL, ∼ 4 μM) are in 50 mM PIPES (pH 6.5), HEPES (pH 7.5), or Tris-HCl (pH 8.5) unless otherwise noted. K^+^ and Mg^2+^ denote 200 mM KCl and 5 mM MgCl_2_, respectively. (a) Crescentin_ΔL1_ in 5 mM buffer. Single 9-nm-wide filaments are denoted with arrows, and filaments with approximate widths of 18 nm (arrowhead) or 27 nm (asterisk) are marked. (b) Crescentin_ΔL1_ in 50 mM buffer. The arrowhead denotes an 11 nm-wide filament. (c) Crescentin_ΔL1_ in the presence of 200 mM K^+^. Arrowheads mark globular structures with approximate diameters of 20–30 nm. (d) Crescentin_ΔL1_ in the presence of 5 mM Mg^2+^. The arrow marks a 50 nm-wide structure. (e) Crescentin_ΔL1_ at pH 7.5 with 5 mM Mg^2+^. A structure with 80 nm width is marked (arrow). (f) Crescentin_ΔN27_ in 50 mM buffer. Arrows denote 8–9 nm-wide filaments (also in inset), the arrowhead denotes a 16 nm-wide filament, and the asterisk denotes a 24 nm-wide filament. (g) Crescentin_ΔN27_ in the presence of 5 mM Mg^2+^. Arrow in inset denotes a 9 nm-wide filament, and the arrowhead denotes a 17 nm-wide filament. (h) Crescentin_ΔN27_ in the presence of 200 mM K^+^. Arrow denotes a 22 nm-wide filament. (i) Crescentin_SAT_. A single 9 nm-wide filament (inset, arrowhead) and 40 nm-wide bundles (arrows) are denoted. Bundled areas showed an axial repeat of 57.1 ± 0.7 nm (*n* = 25). (j) Crescentin_SAT_ in the presence of 5 mM Mg^2+^. The arrowhead marks a 10 nm-wide filament. (k) Crescentin_SAT_ at pH 7.5 in the presence of 5 mM Mg^2+^. The paracrystalline structures have variable width and an axial repeat of 58.7 ± 0.3 nm (*n* = 30). Inset shows a detail of the striations. (l) Crescentin_ΔT_. Single filaments (arrowheads, inset) displayed widths of 17–20 nm. (m) Crescentin_ΔT_ in the presence of 5 mM Mg^2+^. Tapered paracrystalline bundles had an axial repeat of 55.6 ± 0.6 nm (*n* = 30). (n) Crescentin_ΔT_ at pH 7.5 in the presence of 5 mM Mg^2+^.

Surprisingly, TEM of crescentin_ΔL1_ revealed filamentous structures rather than amorphous aggregates. In weak 5 mM buffer at pH 6.5, filaments of varying width were observed, with single 9 nm filaments (Fig. 6a, arrows) as well as filaments with twice or three times that width (Fig. 6a, arrowhead and asterisk, respectively). Filaments were often much shorter than those formed by wild-type crescentin (Fig. 6a, asterisk; compare to Fig. 5b). In 50 mM buffer at pH 6.5, tapered structures with varying width were common, with the narrowest filaments about 10–12 nm wide (Fig. 6b, arrowhead). In the presence of 200 mM KCl, crescentin_ΔL1_ formed large, rough, aggregative structures that appeared to contain globular particles with 20–30 nm diameter (Fig. 6c, arrowheads). Such structures would be consistent with the insolubility of crescentin_ΔL1_ in 200 mM K^+^ (Fig. 5) but are clearly dissimilar to normal filamentous networks. With 5 mM Mg^2+^ ions, broad, tapered structures formed (Fig. 6d), consistent with enhanced lateral association mediated by the divalent cations. With Mg^2+^ at pH 7.5, similar broad structures predominated (Fig. 6e).

Among the other mutants, crescentin_ΔN27_, crescentin_SAT_, and crescentin_ΔT_ were notable for their high sensitivity to 200 mM K^+^ ions, which substantially reduced their solubility at pH 6.5 (Fig. 5). Crescentin_ΔN27_ was also extremely soluble in weak (5 mM) buffer at pH 6.5, and was the most soluble mutant, along with crescentin_SAT_, in Mg^2+^ at pH 7.5 (Fig. 5). The heightened sensitivity of crescentin_ΔN27_, crescentin_SAT_, and crescentin_ΔT_ to potassium may help explain the relatively high frequency of cells with diffuse crescentin localization in these three mutants (Fig. 3). Cytoplasmic K^+^ ions could lower the favorability of assembly to the point where only a fraction of cells successfully assemble a crescentin structure (see Discussion).

Consistent with the first 27 amino acids of crescentin primarily functioning in membrane attachment of crescentin structures rather than in filament formation in vivo [Cabeen et al.,^2009^], purified crescentin_ΔN27_ assembled into filaments largely indistinguishable from wild-type. At pH 6.5, single crescentin_ΔN27_ filaments of 8–9 nm width were commonly observed (Fig. 6f, arrows), as well as bundles with double or triple this width (Fig. 6f, arrowhead and asterisk, respectively) and larger loose bundles. Similar to wild-type crescentin, addition of 5 mM Mg^2+^ ions at pH 6.5 resulted in smooth but thicker crescentin_ΔN27_ filaments of about 17 nm (Fig. 6g, arrowhead), though single 9-nm filaments were still visible (Fig. 6g, inset, arrow). In the presence of 200 mM K^+^ ions, crescentin_ΔN27_ formed rough, branched structures with a 22-nm width (Fig. 6h, arrow). The filaments observed for crescentin_ΔN27_ are in accord with earlier results from our laboratory using His-tagged crescentin_ΔN27_ assembled at pH 7.0 [Ausmees et al.,^2003^]. The previous analysis used crescentin_ΔN27_, instead of wild-type crescentin as we originally assumed, because of a mis-annotated start site in the genome sequence database; we have since confirmed the correct start site using mass spectrometry (Supporting Information Fig. S1) and have updated the public database. In any case, loss of the N27 region of crescentin does not substantially alter filament assembly properties in vitro.

At pH 6.5, crescentin_SAT_ formed structures that were characterized by axial striations displaying a 57.1 ± 0.7 nm repeat (*n* = 25; Fig. 6i, inset) most often in filament bundles. Individual filaments with 9 nm width were also visible (Fig. 6i, inset, arrowhead), as were bundles ∼ 40 nm in width (Fig. 6i, arrows). No striations were seen in the smooth crescentin_SAT_ filament bundles polymerized in the presence of magnesium ions (Fig. 6j). However, at pH 7.5 in the presence of Mg^2+^, spike-like paracrystals were the predominant species, with an axial repeat of 58.7 ± 0.3 nm (*n* = 30; Fig. 6k, inset). The thickness of the structures suggests that a possible function of the stutter is to prevent excessive bundling of crescentin filaments. Such paracrystalline structures have been observed in the presence of divalent cations for truncation mutants of a eukaryotic IF protein, glial fibrillary acidic protein [Quinlan et al.,^1989^; Stewart et al.,^1989^] and for tailless *Xenopus* vimentin [Eckelt et al.,^1992^].

Crescentin_ΔT_ formed long, smooth filaments at pH 6.5 that were similar in appearance to wild-type crescentin filaments, except that the narrowest structures observed were 17–20 nm wide (Fig. 6l, arrowheads) rather than 8–10 nm. Addition of Mg^2+^ ions at pH 6.5 caused filament bundling and the appearance of striations with 55.6 ± 0.6 nm spacing (*n* = 30; Fig. 6m). The crescentin_ΔT_ filament bundles often appeared tapered at their ends. With magnesium at pH 7.5, no striations were visible in the filamentous structures with varying width (Fig. 6n). The similarity of crescentin_ΔT_ to wild-type in vitro is consistent with it being the most functional of all the mutants.

## Discussion

In this study, we used the simple and tractable organism *C. crescentus* with two goals in mind. First, we aimed to discover whether the IF-like domains of crescentin are in fact important for its structure and function. Second, we analyzed different domain mutants to learn whether specific domains have distinct structural or functional roles. For this, we utilized the advantages of our bacterial model system—genetic tractability, a quantifiable cell curvature phenotype and a well-defined cellular localization pattern. Deletion or abrogation of either head subdomain, linker 1, rod, stutter, or tail all had profound effects on crescentin function in vivo, indicating that many elements of the IF-like organization of crescentin are indeed functionally important. Moreover, each mutation had a distinct effect, in accord with different domains having different roles.

Based on our data, we suggest the following functions for each element. Mutants lacking the N28-79 head subdomain and rod domains display no structure in vivo, suggesting a fundamental role for these elements in filamentous structure assembly. Linker L1 may play the opposite role, preventing the runaway polymerization of crescentin that disrupts its normal filamentous cellular localization and function. It may fulfill this function simply by providing an interruption in the coiled-coil rod domain. Linker L1 also appears to be like the stutter in that both these regions lower the propensity for lateral association, most apparent in vitro by the broad structures formed by crescentin_ΔL1_ and paracrystal formation by crescentin_SAT_. It is clear that crescentin_ΔN27_, crescentin_SAT_, and crescentin_ΔT_ are capable of assembling into long filaments in vitro. Therefore, the observations that crescentin_SAT_ and crescentin_ΔT_ typically form only short structures in cells, and that crescentin_ΔN27_ and crescentin_SAT_ often show diffuse localization, are not best explained by a structural defect. Instead, our in vitro data suggest that the tail, stutter, and N27 regions stabilize crescentin against disassembly in the presence of K^+^ ions. This is not a trivial role, as several studies have estimated *E. coli* cytoplasmic potassium concentration at about 200 mM [Prigent-Combaret et al.,^1999^; Outten and O'Halloran,^2001^; Shabala et al.,^2009^]. Assuming a similar concentration in *C. crescentus*, this level would be adequate for a substantial inhibition of in vivo crescentin assembly in these three mutants. Cytoplasmic K^+^ ions may favor disassembly of these mutants so that full elongation of crescentin structures is very rare, perhaps explaining the high frequency of diffuse localization or short structures in cells producing these mutants.

Collectively, our study suggests that in order to function, crescentin must meet multiple criteria. First, the protein must assemble into a structure, a function that appears to require the N28-79 and rod domains. Next, it must be able to assemble even in the presence of K^+^ ions, a function to which the N27, stutter, and tail regions contribute. However, crescentin must not polymerize excessively or bundle too much—the L1 linker and the stutter assist this function. Finally, crescentin must be able to associate with the membrane, a function served by the N27 region.

IF proteins are found in all animal cells, and the Human Intermediate Filament Database (www.interfil.org/diseases) lists 90 human diseases that have been linked to mutations in IF genes. Still, IFs are the least understood of the three major eukaryotic cytoskeletal systems. Research efforts have been hampered by the large number of IF proteins in mammalian systems, the possible functional redundancy between specific IFs, and the lack of powerful genetic model systems in which to study them. Our study shows that crescentin not only bears but requires its characteristic IF features—head, rod, linker 1, stutter, and tail—for structure and function. This structural similarity to IF proteins, combined with the IF-like dynamic and mechanical properties of crescentin in vitro and in vivo [Cabeen et al.,^2009^; Charbon et al.,^2009^; Esue et al.,^2010^], establishes crescentin as a valuable bacterial model for the study of a broad family of IF proteins that we propose includes both eukaryotic and prokaryotic members.

By the term “family,” we mean a group of proteins that shares a characteristic set of IF-like biochemical, mechanical, and cytoskeletal properties deriving from a particular protein organization. We do not imply any particular evolutionary relationship between crescentin and animal IF proteins, as we lack sufficient data to make definitive statements. While it seems clear that both crescentin and animal IF proteins derive from a bacterial coiled-coil-rich protein ancestor, how IF-like domain organization arose in each case remains obscure. It is formally possible that it arose in bacteria, further evolved into a more constrained structural organization in eukaryotes, and was then lost in many species. Alternatively, IF-like organization may have independently evolved in bacterial and animal cells through convergent evolution of a basic coiled-coil motif. Finally, we cannot rule out lateral gene transfer from animal cells to bacteria, although the current aquatic, nutrient-poor ecological niche of *C. crescentus* does not suggest close interactions with animal cells.

Importantly, this IF family is a subset of a coiled coil-rich protein superfamily, and eukaryotic IF proteins are a further, more constrained, subset. In this way, the IF family resembles the actin family, which is a subset of a nucleotide-binding protein superfamily, and includes both the divergent bacterial actin proteins and the highly conserved eukaryotic actin [Bork et al.,^1992^; Derman et al.,^2009^]. There are some variations between crescentin and eukaryotic IF proteins, in coiled-coil motif length and in amino acid sequences at either end of the rod domain, and these differences parallel the distinction between bacterial and eukaryotic tubulin and actin homologs. Just as bacterial actins and tubulin homologs display a core set of motifs in order to function, crescentin contains a core set of IF protein features that allow its function. We speculate that during evolution, the actin, tubulin, and IF motifs have been selected for their utility and versatility as cytoskeletal elements. It will surely be interesting to explore how bacteria use other variations of the IF architecture [Izard,^2006^; Bagchi et al.,^2008^] to fulfill different cellular roles.

## Abbreviations

GFP: green fluorescent protein
HEPES: 4-(2-hydroxyethyl)-1-piperazineethanesulfonic acid
IF: intermediate filament
MWCO: molecular weight cut-off
LB: lysogeny broth
PAGE: polyacrylamide gel electrophoresis
PIPES: piperazine-N,N′-bis(2-ethanesulfonic acid)
SDS: sodium dodecyl sulfate
TEM: transmission electron microscopy.
