# Supplementary material for: The domain organization of the bacterial intermediate filament-like protein crescentin is important for assembly and function
Source: Cytoskeleton (Hoboken). 2011 Feb 28;68(4):205–19. doi: 10.1002/cm.20505 (PMC3087291; doi:10.1002/cm.20505)
Supplement: Supplementary file 1 [file cm0068-0205-SD1.pdf]

Supplementary Text

Functional analysis of intermediate filament protein domain organization of crescentin

M. Cabeen, H. Herrmann and C. Jacobs-Wagner

Contents:

Supplementary Figure S1

Strain and plasmid modes of construction

PCR primer sequences

References

a

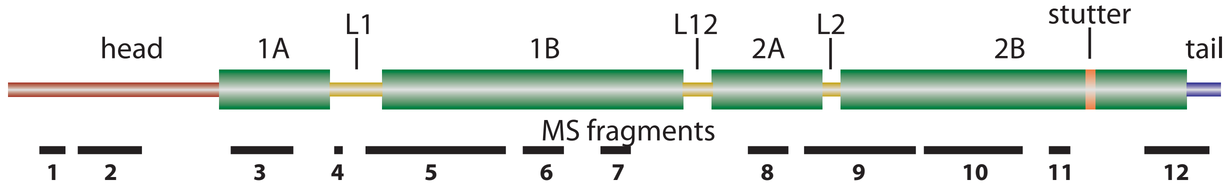

b

annotated start site

1 MRLLSKNSRE TKNGKPTVLG DEARAEAMQH QIESTQAIGQ RYETIHGGLD SIGRVMEHLK

61 AIEPLIAEIR GPVSQEFEAR RAEHAELIAV RANLDQAQRQ IALIQAEERE VSARLAAAET

121 ALGESDARRQ TQDAALEDNA LEIDRLRNAL LQSDLKVSSL DASLRDATAR IEHLVQDVEG

181 LRVQAQDIDA RRGDAEAALA RANQDNALLG EEAATLKKRV DQAGLDLARL SRIETDLEAQ

241 LAAERARVQA VENALAAHQA DSGRTIRGLE SQVEANRAEI SALQTRLETA TGRADKLEEM

301 NGQISARLAD SSAQQKAVER RAGDLNVALE RALDRIRALE EEADGLRQRH AGVDTARATA

361 IERADQLAKS AVAQEKALKR AEERAQQLRA RLDAMQEAQD QVRRDHEAKI AELQATIERL

421 TSEAALAEGA LEAARRDRSR LQMALLGASD GDVAASA

Figure S1

a. Schematic of crescentin domain organization. Coiled coil-forming regions are indicated by thick green bars; linkers L1, L12, L2, head, tail, and stutter are labeled. Fragments of crescentin protein identified by mass spectrometry are indicated below the diagram. **b.** Detected tryptic fragments of crescentin-FLAG, immunoprecipitated from CJW932 (CB15N *creS*::pJM21creS-flag), are indicated in red on the full crescentin sequence.

Strain and plasmid modes of construction

C. crescentus strains

CJW1231

Plasmid pMR20creS-tc was into CB15N.

CJW1474

Plasmid pMR20creSΔT was electroporated into CB15N.

CJW1475

Plasmid pMR20creSΔT was electroporated into CB15N Δ*creS* (LS3812).

CJW1478

Plasmid pMR20creSΔR-tc was electroporated into CB15N.

CJW1518

Plasmid pMR20creSSAT-tc was electroporated into CB15N Δ*creS* (LS3812).

CJW1520

Plasmid pMR20creSΔL1-tc was electroporated into CB15N.

CJW1521

Plasmid pMR20creSΔL1-tc was electroporated into CB15N Δ*creS* (LS3812).

CJW1522

Plasmid pMR20creSSAT-tc was electroporated into CB15N.

CJW1535

Plasmid pMR20creSΔN28-79-tc was electroporated into CB15N.

CJW1536

Plasmid pMR20creS ΔN28-79-tc was electroporated into CB15N Δ*creS* (LS3812).

CJW2862

Plasmid pMR20creSΔR-tc was electroporated into CB15N Δ*creS* (LS3812).

***E. coli* strains**

CJW1353, 1659, 2045, 2054, 3663

BL21 (DE3) was transformed with the plasmids listed in the strain table by electroporation.

Plasmids

pET29acreS

creS was excised from pBGENT-NdecreS (**CJW1629**) with NdeI/HindIII and ligated into pET29a cleaved with the same enzymes.

pET29acreSΔN27

creSΔN27 was excised from pKS-NdecreSΔN27 (**CJW912**) with NdeI/HindIII and ligated into pET29a cleaved with the same enzymes.

pET29acreSΔL1

Replaced the wild-type allele with the *creS*$_{\Delta L1}$ allele by replacing an NcoI/StuI fragment from pHL23PxylcreS with the corresponding ΔL1 fragment, generating pHL23PxylcreSΔL1. Excised *creS*$_{\Delta L1}$ from this plasmid with NdeI/HindIII and ligated into pET29a cleaved with the same enzymes.

pET29acreSSAT

Ligated the 5' half of *creS*, cleaved from pBGENT-NdecreS (**CJW1629**) with NdeI/StuI, with the 3' half of *creS*$_{SAT}$, cleaved from pHL23creSSAT with StuI/HindIII, into pET29a cleaved with NdeI/HindIII.

pET29acreSΔT

Cleaved *creS*$_{\Delta T}$ from pHL23creSΔT with NdeI/HindIII and ligated into pET29a cleaved with the same enzymes.

pMR20creSΔN28-79-tc

The *creS* gene was amplified with primers 219 and 61, which introduced an NdeI site and a start codon immediately preceding codon 80 of the *creS* coding sequence and a HindIII sequence at the 3’ end of *creS*. About 0.5kb of the *creS* 5’ UTR, presumably containing P_{creS} and extending to codon 27 of the *creS* coding sequence, was amplified with primers 60 and 220, introducing an NdeI site at the 3’ end and an EcoRI site at the 5’ end. The former PCR product was cleaved with NdeI/HindIII, and the latter with EcoRI/NdeI; the cleaved products were ligated together into pKS cleaved with EcoRI/HindIII (forming **pKScreSΔN28-79**). An EcoRI/StuI fragment containing the promoter and 5’ third of *creS*$_{\Delta N28\text{-}79}$ was cleaved from pKScreSΔH and ligated with a StuI/HindIII fragment from pKScreS-tc into pMR20 cleaved with EcoRI/HindIII.

pMR20creSΔR-tc

The pKScreS plasmid was amplified with primers 246 and 383, representing the 5’ UTR up to the fifth heptad repeat of coil 1A, and with primers 384 and 247, representing the last 5 heptad repeats of coil 2B to the end of the cloned sequence. These amplicons were then used in a second PCR to self-prime with 20 nt of overlapping sequence, and were amplified with the nested primers 121 and 269, adding an NdeI site before codon 28 and sequence encoding a tetracysteine tag and HindIII site at the 3’ end. This product was cleaved with NdeI and HindIII and triple-ligated with an EcoRI/NdeI fragment containing the 5’ UTR of *creS* up to codon 27 into pKS cleaved with EcoRI/HindIII (creating **pKScreSΔR**). The insert was then sequenced. This strategy resulted in an amino acid substitution at codon 27 due to the inserted NdeI site; to remove this, pKScreSΔR was cleaved with NcoI/HindIII (retaining the ΔR deletion) and ligated with an EcoRI/NcoI fragment from pKScreS into pHL23 cleaved with EcoRI and HindIII. The fragment was then cut from pHL23 with EcoRI/HindIII and ligated into pMR20 cut with the same enzymes.

pMR20creSΔT

The pKScreS plasmid was amplified with primers 60 and 382, the product of which included the 5' UTR (~500bp, thus including the putative promoter) and replaced codon 445 with a TAA stop codon, thus truncating the last 13 amino acids of the protein, the putative non-helical tail. The primers introduced 5' EcoRI and 3' HindIII; the PCR product was cleaved with these enzymes, ligated into pHL23, and sequenced. The fragment was then cleaved from pHL23 with EcoRI/HindIII and ligated into pMR20 cut with the same enzymes.

pMR20creSSAT-tc

The pKScreS plasmid was amplified with primers 246 and 385, representing the 5' UTR up to codon 405 of the *creS* coding sequence and adding 9 nucleotides encoding Ser-Ala-Thr, and with primers 386 and 247, adding the complement to the nucleotides encoding Ser-Ala-Thr and amplifying codon 406 to the end of the cloned sequence. These amplicons were then used in a second PCR to self-prime with 9 nt of overlapping sequence (coding from Ser-Ala-Thr; SAT), and were amplified with the nested primers 114 and 269 amplifying most of the $creS_{SAT}$ sequence and adding and sequence encoding a tetracysteine tag and HindIII site at the 3' end. This product was blunt-end cloned into pHL23 cut with EcoRV, sequenced, then cleaved from this plasmid with SalI and HindIII and triple-ligated with an EcoRI/SalI fragment containing the 5' portion of *creS* from pKScreS into pHL23 cleaved with EcoRI and HindIII. The fragment was then cut from pHL23 with EcoRI/HindIII and ligated into pMR20 cut with the same enzymes. According to the MARCOIL algorithm (Delorenzi and Speed 2002), the SAT insertion completely restores the coiled-coil probability that is disrupted in the wild-type sequence.

PCR Primers

60 5' CTTCGCTTGAATCGGCTCGCCCA

61 5' CGATGACCATCGTCCTGGCCGA

114 5' CGAAACGGCGATCGACCGCCTGCGCAACGC

121 (NdeI) 5' TCACATATGCAGCACCAGATCGAGTCCA

219 (NdeI) 5' ACATCACATATGCGTCGCGCCGAGCACGCCGAGCT

220 (NdeI) 5' ACTATCCATATGCTCGGCGCGAGCTTCATCAC

246 5' GTAAAACGACGGCCAGT

247 5' CAGGAAACAGCTATGAC

269 (HindIII) 5' ACAAAAGCTTTTAACAACATCCTGGACAACAGGCGCTCGCGGCCACGTCGCCG

382 (HindIII) 5' ACAAAAGCTTTTAGGCCATCTGCAGGCGTGAGCGG

383 5’ GCTCGGCGATGCGGGCGGAAACCTCGCGTTCCTCG

384 5’ TTCCGCCCGCATCGCCGAGCTGCAGGCGACCATCG

385 5’ GGTAGCTGAATCGCGGCGAACCTGGTCTTGCG

386 5’ TCAGCTACCCACGAGGCGAAGATCGCCGAGC

References

Delorenzi M, Speed T. 2002. An HMM model for coiled-coil domains and a comparison with PSSM-based predictions. Bioinformatics 18(4):617-25.
